# Computational Identification of a Putative Allosteric Binding Pocket in TMPRSS2

**DOI:** 10.3389/fmolb.2021.666626

**Published:** 2021-04-30

**Authors:** Jacopo Sgrignani, Andrea Cavalli

**Affiliations:** ^1^Institute for Research in Biomedicine, Università della Svizzera Italiana, Bellinzona, Switzerland; ^2^Swiss Institute of Bioinformatics, Lausanne, Switzerland

**Keywords:** TMPRSS2 protein, molecular modeling, allosteric pocket, docking, MD simulation

## Abstract

Camostat, nafamostat, and bromhexine are inhibitors of the transmembrane serine protease TMPRSS2. The inhibition of TMPRSS2 has been shown to prevent the viral infection of severe acute respiratory syndrome coronavirus 2 (SARS-CoV-2) and other viruses. However, while camostat and nafamostat inhibit TMPRSS2 by forming a covalent adduct, the mode of action of bromhexine remains unclear. TMPRSS2 is autocatalytically activated from its inactive form, zymogen, through a proteolytic cleavage that promotes the binding of Ile256 to a putative allosteric pocket (A-pocket). Computer simulations, reported here, indicate that Ile256 binding induces a conformational change in the catalytic site, thus providing the atomistic rationale to the activation process of the enzyme. Furthermore, computational docking and molecular dynamics simulations indicate that bromhexine competes with the N-terminal Ile256 for the same binding site, making it a potential allosteric inhibitor. Taken together, these findings provide the atomistic basis for the development of more selective and potent TMPRSS2 inhibitors.

## Introduction

Since the early days of the pandemic coronavirus disease 2019 (COVID-19) started from the Chinese city of Wuhan, Hubei province, in December 2019, many reports highlighted the crucial role of transmembrane serine protease 2 (TMPRSS2) in the spread and progression of the viral infection (Hoffmann et al., 2020; Sungnak et al., 2020). TMPRSS2 has been identified as one of the proteases responsible for the proteolytic priming of SARS-CoV-2 spike protein which leads to the release of the fusion peptide. In addition to that, TMPRSS2 has been put in relation with the spread of other viruses, such as influenza A viruses, severe acute respiratory syndrome coronavirus 2 (SARS-CoV), and Middle East respiratory syndrome coronavirus (MERS-CoV), and it has been studied as a potential therapeutic target for prostate cancer therapy (Lucas et al., 2014; Shen et al., 2017). Finally, as TMPRSS2 expression is regulated by the androgen receptor, it has been hypothesized that its crucial role in the viral infection might help explain why males have more frequently severe complications and a worse clinical outcome than females and if androgen deprivation therapy (ADT) can have a protective effect against SARS-CoV-2 infection (Montopoli et al., 2020). These observations stimulated intense investigations, and the number of papers with the TMPRSS2 keyword in the title indexed in PubMed during 2020 raised from an average of 80–100/year to 601.

TMPRSS2 is a membrane protein belonging to the type II transmembrane serine protease (TTSP) family. It is functionally classified as a trypsin-like protease (TLP). Like other serine proteases, TMPRSS2 cleaves peptide bonds that are present after positively charged residues (lysine or arginine), and its enzymatic activity depends on the presence of a catalytic triad formed by His296, Asp345, and Ser441. The catalytic selectivity is achieved with the presence of a negatively charged Asp residue at the bottom of a cavity usually indicated as “S1 specificity pocket” (Laporte and Naesens, 2017; Singh et al., 2020).

**FIGURE 1:**
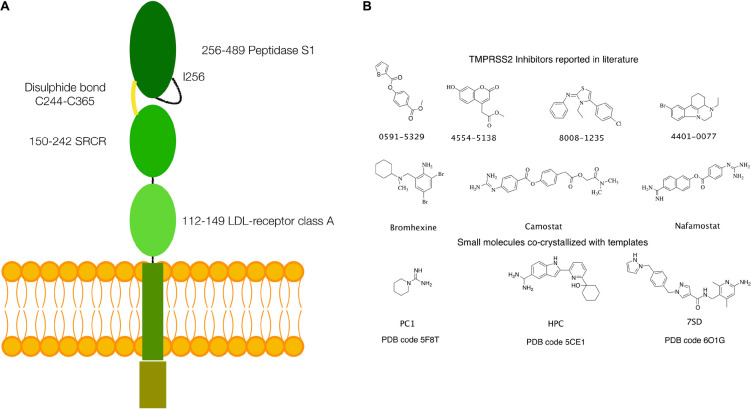
**(A)** Schematics of the structure of TMPRSS2. **(B)** Small molecules with inhibitory activity on TMPRSS2 reported in the literature and small molecules or fragments co-crystallized in the S1 specificity pocket in the templates used for homology modeling.

Structurally, TPMRSS2 is characterized by the presence of a cytoplasmic N-terminal domain, a transmembrane helical domain, and three extracellular domains: low-density lipoprotein (LDL)-receptor class A domain, scavenger receptor cysteine-rich (SRCR) domain, and the peptidase S1 domain, also called serine protease domain (SPD) (Figure 1A).

An autocatalytic cleavage between Arg255 and Ile256 activates the 492-residue long TMPRSS2 zymogen. This modification enables the binding of Ile256 into a putative allosteric pocket (A-pocket), which induces a conformational rearrangement of the catalytic site (Bertram et al., 2010). After the cleavage, membrane TLPs, such as TMPRSS2, remain bound to the transmembrane N-terminal domains by a conserved disulfide bond, although a small fraction of the protein can be detected into the extracellular milieu (Szabo et al., 2003; Pászti-Gere et al., 2016; Shen et al., 2017).

Two different species are reported in the literature, one with a mass of ∼55 kDa that corresponds to the full-length protein and one of ∼30 kDa which represents the SPD released in the extracellular space if the disulfide bond is not formed (Afar et al., 2001; Chen et al., 2010).

To date, no atomistic structure of the entire TMPRSS2, or the SPD, is available. However, important information can be derived from the structure of homologous proteins such as matriptase, DESC1, and several kallikreins.

Several inhibitors of TMPRSS2 have been identified in the last years. These include organic compounds such as camostat, nafamostat, and bromhexine (BH) (Figure 1B) and peptidomimetics (Meyer et al., 2013; Lucas et al., 2014; Shen et al., 2017; Bestle et al., 2020; Hoffmann et al., 2020; Zang et al., 2020). Of particular note is BH, a component of widely used medicaments against respiratory disorders characterized by viscid or excessive mucus. In fact, following the report of a selective TMPRSS2 inhibition by Lucas et al. (2014), the use of BH for the prevention and therapy of the SARS-CoV-2 infection has been hypothesized (Depfenhart et al., 2020; Habtemariam et al., 2020; Maggio and Corsini, 2020). However, to date, only a limited number of clinical trials have been carried out, and their results remain inconclusive (Ansarin et al., 2020; Li et al., 2020).

In this work, we used computational and experimental methods, such as homology modeling, molecular docking, molecular dynamics (MD), and microscale thermophoresis (MST), to investigate the structure and dynamics of TMPRSS2 and clarify its activation mechanism and the interaction with various inhibitors at an atomistic level of details. We focused, in particular, on the differences in the mode of action of camostat/nafamostat and BH. In fact, while camostat and nafamostat inhibit TMPRSS2 by forming a covalent adduct, the mode of action of BH remains unclear.

Besides the generation of a reliable model of the TMPRSS2 catalytic domain, the results of our investigations confirmed that both camostat and nafamostat are competitive inhibitors efficiently binding the active site. Contrarily, they indicated that the binding of BH to the active site is unlikely, leading us to the identification of a putative allosteric binding pocket.

## Materials and Methods

### Homology Modeling

An atomistic model of the SPD of TMPRSS2 (UniProt code O15393), covering residues from Ile256 to Gly492, was generated by homology modeling. The most suitable templates were identified using the SWISS-MODEL webserver (Waterhouse et al., 2018). This search provided three templates [Protein Data Bank (PDB) codes: 5F8T, 5CE1, and 6O1G], having a sufficient degree of similarity (between 38 and 41%), thus well-suited for an accurate model generation (Xiang, 2006; Cavasotto and Phatak, 2009; Sgrignani et al., 2018). The alignment between the target sequence and the templates was performed using the Prime-STA algorithm, included in the Schrodinger suite for molecular modeling (Schrodinger Suite 2020-1). This algorithm, in addition to the sequence alignment, considers secondary structure matching, providing better alignments also in poorly conserved regions. Next, 10 models were generated for each of the three templates using PRIME, keeping small ligand molecules, such as piperidine-1-carboximidamide (PC1), 2-[6-(1-hydroxycyclohexyl)pyridin-2-yl]-1H-indole-5-carboximidamide (HCP), and N-[(6-amino-2,4-dimethylpyridin-3-yl)methyl]-1-({4-[(1H-pyrazol-1-yl)methyl]phenyl}methyl)-1H-pyrazole-4-carboxamide (7SD) (Figure 1), bound to the protein active site in the templates 5F8T, 5CE1, and 6O1G, to preserve their respective conformations.

Finally, the models (subsequently indicated as M-5F8T, M-5CE1, and M-6O1G) with the lowest OPLS3e (Harder et al., 2016) potential energy after minimization were selected for the subsequent calculations.

### Molecular Dynamics Simulations

Atomistic models were prepared for MD simulation with the following protocol: (1) the PROPKA program was used to assign the residue protonation state at a reference pH of 7.4 (Olsson et al., 2011) and (2) the structures were solvated in a box of water with a minimal distance from the protein surface of 10 Å. A proper number of counterions were added to the systems to ensure charge neutrality. All the non-solvent molecules were parametrized using the OPLS3 (Harder et al., 2016) force field, while TIP3P model (Jorgensen et al., 1983) was used for water molecules.

Before the MD production runs, the following simulation protocol was used to equilibrate the systems: (1) Brownian dynamics was run for 100 ps in an NVT ensemble (*T* = 10 K) applying harmonic restraints on solute heavy atoms (force constant 50 kcal/mol/Å^2^); (2) NVT (*T* = 10 K) MD simulation of 12 ps in NVT ensemble conserving the same restraints applied in (1); (3) NPT (*T* = 300 K and *P* = 1 atm) MD simulation (12 ps) conserving the same restraints applied in (1); and (4) NPT (*T* = 300 K and *P* = 1 atm) MD simulation (24 ps) without restraints. The pressure and the temperature were fixed at 300 K and 1 atm by the Martyna–Tobias–Klein barostat (Martyna et al., 1994) and the Nosé–Hoover chain thermostat (Martyna et al., 1992), respectively. All the simulations were run using GPU accelerated DESMOND code. A summary of the simulations run in this work is reported in Table 1.

**TABLE 1:** Summary of the performed molecular dynamics.

**Description of the system**	**Number of independent simulations**	**Simulation length (ns)**	**Ligand**
M-5F8T	3 + 3 (His296 protonated on the ε nitrogen)	250	
M-5CE1	3	250	
M-6O1G	3	250	
M-5F8T	1 + 1 (His296 protonated on the ε nitrogen) for both camostat and nafamostat	500	Camostat and nafamostat
M-5F8T	3	2 × 1,000 1 × 500 (the complex decomposed)	BH in Site_1
M-5F8T	1	100	BH in Site_2
Apo-C-M-5F8T	1	1,000	
Apo-M-5F8T	1	1,000	
C-M-5F8T	3	500	(R)-BH in the A-pocket
C-M-5F8T	3	500	(S)-BH in the A-pocket
C-M-5F8T	3	500	(R)-BH in the A-pocket (IFD docking)
C-M-5F8T	3	500	(S)-BH in the A-pocket (IFD docking)

**The models from different templates were indicated as M-5F8T, M-5CE1, and M-6O1G. The suffix C indicates the models where the first two residues (Ile256 and Val257) were deleted.**

Root mean square deviation (RMSD), root mean square fluctuation (RMSF), and radius of gyration (Rg) analysis were computed using Maestro (Schrodinger Suite 2020-1). Cluster analysis was performed with the program TTClust (Tubiana et al., 2018), focusing on residues belonging to the catalytic site, namely, Cys281, Thr293, Ala294, Ala295, His296, Cys297, Val298, Glu299, Tyr337, Asp338, Ser339, Lys342, Asn343, Ans344, Asp345, Ile346, Ala347, Met424, Cys437, Gln438, Asp440, Ser441, Asp458, Thr459, Ser460, Trp461, and Phe480. Contrarily, the analysis of the loop that regulates the access to the S1 specificity pocket was performed considering all the residues between Gly462 and Val473. The optimal number of clusters was automatically determined using the “elbow” method with k-means (Tibshirani et al., 2001).

### Computational Docking of TMPRSS2 Ligands

Computational docking was performed using the software GLIDE (Friesner et al., 2004). The analysis of the structural parameters and the analysis of MD simulations (see section “Results and Discussion”) indicated M-5FT8 as having a higher quality and more stable among the generated models.

In analogy to the previous studies (Amaro et al., 2008, 2018; Sgrignani et al., 2009), to account for target flexibility, snapshots from MD simulations of M-5FT8 were selected using the previously described cluster analysis. In particular, four snapshots were selected from the simulations run with positively charged His296 and four from the simulations with His296 protonated on the ε nitrogen (see also section “Results and Discussion”).

The grids for docking were centered in the geometric center of all the atoms of the three residues forming the catalytic triads (His296, Asp345, and Ser441). A distinct grid file was generated for all selected snapshots.

Contrarily, for the docking of BH in the putative site predicted by Sitemap, the grid was centered using the corresponding sitepoints. In this context, sitepoints are points in a grid, contiguous, or bridged by short gaps in exposed regions, that define the shape of a putative binding site (Halgren, 2007).

All docking calculations were performed using the standard precision (SP) protocol and GlideScore. Furthermore, docking was performed on all selected snapshots, and, finally, the pose with the best GlideScore, together with the receptor, was saved for the analysis and MD simulations.

The structures of the small molecule ligands were prepared with LIGPREP. In the case of BH, the results indicated a protonation of the ternary amino group; therefore, both enantiomeric molecules (S and R) were considered in docking calculations, but only the complex with best GlideScore was used in MD simulations.

**FIGURE 2:**
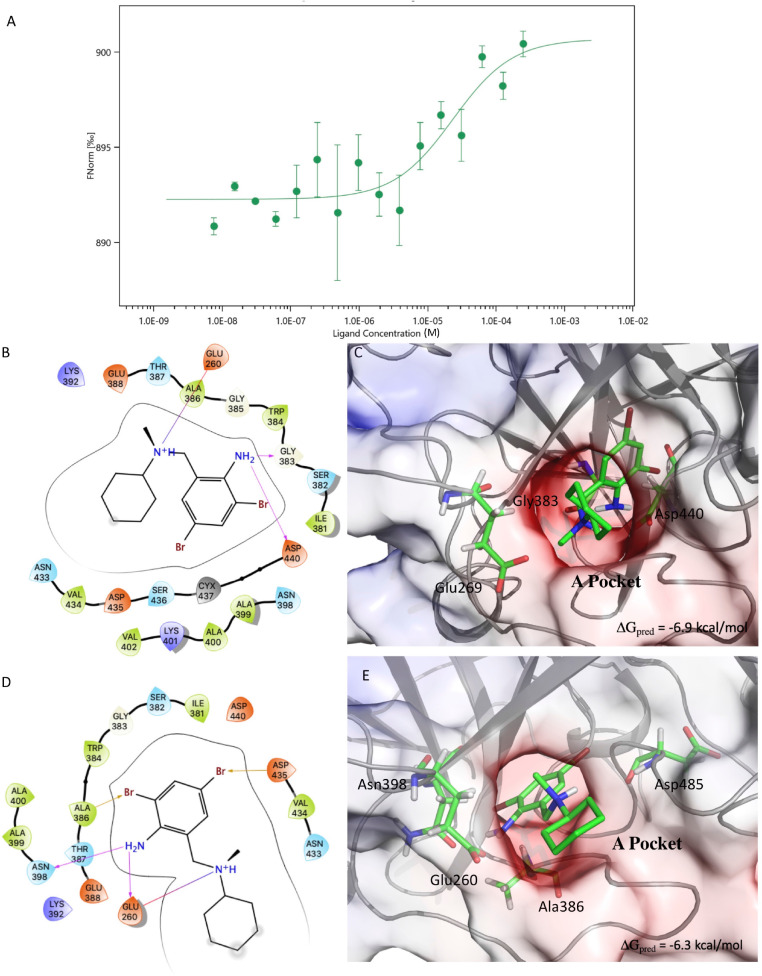
Results of the MST experiments **(A)**. Structures and schemes of the interactions of the S-BH **(B,C)** and R-BH **(D,E)** in complex with C-M-5F8T as resulted from IFD calculations. The protein surface is colored according to the electrostatic potential. The unit of electrostatic potential is kbT/*e* where kb, T, and *e* are the Boltzmann’s constant, absolute temperature, and the charge of an electron, respectively. The ΔG_pred_ values reported in the picture are the GlideScore values obtained from docking calculations. IFD, induced-fit docking; MST, microscale thermophoresis.

Docking of BH in the A-pocket (see section “Results and Discussion” for a definition) was performed using a representative structure of the open and closed conformations (Figures 2B–E) sampled during the MD simulations of C-M-5F8T (see section “Results and Discussion”). In this case, the grid was centered in the COG of the residues Ile381, Ser382, Gly383, Gly385, Ala386, Thr387, Glu388, Asn398, Ala399, Ala400 Asn433, Val434, Asp435, Ser436, Cys437, Asp440, Cys465, and Ala466.

The results of these calculations showed a better GlideScore for the complex in the closed conformation (∼−3.0 kcal/mol vs. ∼−4.8 kcal/mol for open and closed conformations). However, also in this case, the complex with the best GlideScore dissociated during MD simulations.

Regarding this point, it is important to notice that in M-5F8T, the S-pocket is occupied by a small aminoacidic tail. It is, therefore, reasonable to assume that a side chain rearrangement is needed to accommodate different ligands.

Consequently, the docking was performed again using the induced-fit docking (IFD) protocol of GLIDE, with default input parameters. In particular, only the orientations of the side chains of the residues within a distance of 5 Å from the ligand were optimized. Finally, the complex with the lowest IFD score [a specific score that combines GlideScore, Glide_Ecoul energy, and Prime protein conformation energy (Sherman et al., 2006)] was selected as the best model and used in MD simulations.

### Prediction of Putative Allosteric Binding Sites

Several algorithms to detect allosteric pockets in proteins have been developed in the last years (Halgren, 2007, 2009; Yu et al., 2010; Panjkovich and Daura, 2014; Kozakov et al., 2015; Jiménez et al., 2017; Xu et al., 2018; Guarnera and Berezovsky, 2019). Sitemap, proposed by Halgren in 2007 (Halgren, 2007, 2009) and implemented in the Schrodinger suite for molecular modeling, is among the most widely used. Furthermore, it provides a clear assessment of the druggability of the identified pockets.

Consequently, we used this algorithm to investigate the presence of allosteric pockets in both M-5FT8 and C-M-5F8T. All the calculations were performed using default values provided by the Maestro interface (Schrodinger Suite 2020-1). In addition to that, to validate these results, the same structures were analyzed also with other algorithms (PARS, Deepsite, and FTMap), using the respective webservers^1^ ^,2^ ^,3^.

### MST Experiments for the TMPRSS2/BH Binding

The binding affinity between TMPRSS2 and BH was measured by MST. Recombinant human TMPRSS2 (106-492aa, 6xHisTag) was acquired from Cusabio (CSB-YP023924HU) and labeled using a His-tag-specific dye (Monolith His-Tag Labeling Kit RED-tris-NTA (MO-L018), NanoTemper^®^ Technologies GmbH, München, Germany), according to manufacturer instructions. A fixed concentration of the labeled TMPRSS2 (5 nM) was mixed with 16 1:1 serial dilution of BH. MST measurements were performed using premium-coated capillary tubes on a NanoTemper instrument.

BH was first dissolved in DMSO at a 5 mM concentration. In all subsequent experiments, both protein and BH were dissolved in Dulbecco’s Phosphate-Buffered Saline (PBS; D8537, Sigma Aldrich, Saint Louis, MO, United States).

Two independent experiments were performed to compute the Kd values. Data were analyzed with the NanoTemper analysis software MO.Affinity Analysis (v. 2.3). Kd values were obtained fitting compound concentration-dependent changes in normalized fluorescence (Fnorm).

## Results and Discussion

### Homology Modeling of the Serine Protease Domain of TMPRSS2

Considering its relevance for both the drug design and the enzymatic function, we focused our attention on the TMPRSS2 SPD (Ile256 to Gly492).

**FIGURE 3:**
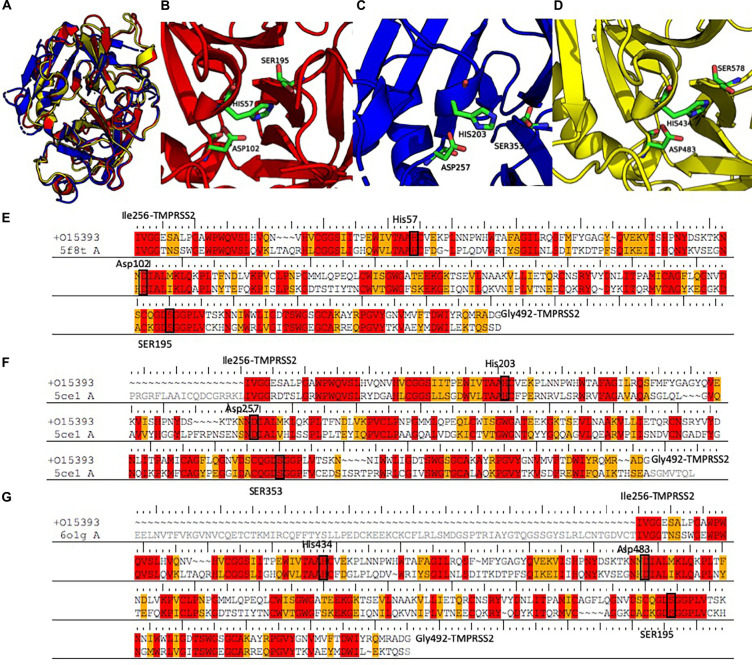
**(A)** Structural alignment between the three selected templates 5F8T (red), 5CE1 (blue), and 6O1G (yellow). Details of the catalytic sites of the PK structures deposited with the PDB code 5F8T **(B)** and 6O1G **(C)** and of the HP structure deposited with the code 5CE1 **(D)**. **(E–G)** Sequence alignments between the three templates and the SPD of TMPRSS2. Identical residues are colored in red; conserved residues (according to the BLOSUM62 scoring matrix) are colored in orange. Abbreviations: PDB, Protein Data Bank; SPD, serine protease domain.

A search performed with the SWISS MODEL webserver identified three very similar structures (Figure 3 and Table 2) as suitable templates to generate TMPRSS2 models: (1) two structures of the human plasma kallikrein (PK), a serine protease that cleaves high-molecular-weight kininogen (HMWK) to generate bradykinin (BK) (Schmaier, 2013) [PDB codes: 5F8T and 6O1G (Partridge et al., 2019), resolution 1.75 and 2.50 Å] and (2) the structure of hepsin (HP), a membrane-bound serine protease able to catalyze protein cleavage after basic amino-acid residues (PDB code: 5CE1, resolution 2.50 Å). In fact, the pairwise RMSD computed using the Cα atoms and the program ALMOST (Fu et al., 2014) is smaller than 0.5 Å.

**TABLE 2:** Summary of the sequence–sequence alignment between the sequence of the serine protease domain of TMPRSS2 and the three selected templates.

**Template PDB code**	**Score**	**Identities (%)**	**Positives (%)**	**Gaps (%)**
5F8T	1,218	41	59	2
5CE1	1,152	39	55	5
6O1G	1,185	41	57	4

**The score is the BLAST bit score. Identities is the percentage of residues that are identical between the sequences. Positives is the percentage of residues that are positive matches according to the similarity matrix (BLOSUM62). Gaps is the percentage of gaps in both the query and homolog as returned by BLAST.**

The sequences of the three selected templates were aligned to TMPRSS2 using the PRIME-STA procedure (Figures 3E–G), and 10 models were generated starting from each template. Finally, the model with the lowest potential energy was selected from the three different groups. As expected, all the three models were very similar, with a pairwise Cα − RMSD below 0.5 Å. Furthermore, visual inspection of the three structures confirmed the similarity between all models, with the exception of the region between Tyr322 and Ser333. In fact, while in the two models derived from PK structures (M-5F8T and M-6O1G), this region is a β-sheet, in the model form HP structure (M-5CE1), it is modeled as a long loop. This is not surprising because in the sequence–sequence alignment between HP and TMPRSS2 used for model generation, this region is characterized by the insertion of three amino acids.

The quality of the models was evaluated with the Protein Structure Quality viewer implemented in Maestro, computing structural parameters widely used in the evaluation of homology models (Sgrignani et al., 2009) and by the PROSA-Web server (Wiederstein and Sippl, 2007; Table 3). This analysis did not show any critical points for all generated models. Nevertheless, the number of violations of the allowed regions in the Ramachandran plot, and other violations from the ideal structural parameters were higher for the models generated using 5CE1 and 6O1G.

**TABLE 3:** Results of the structure quality evaluation.

**Model name**	**Ramachandran violation**	**RMS bond dev.**	**RMS angle dev.**	**Backbone**	**Sidechains**	**Peptide planar dev.**	**Sidechains planar dev.**	**Torsion planar dev.**	**Z-score**
M-5F8T	9	0.020	2.21	5	17	5.80	0.007	1.13	−6.82
M-5CE1	15	0.022	2.27	11	25	6.36	0.008	1.18	−7.12
M-6O1G	19	0.021	3.05	18	23	6.65	0.010	1.45	−6.47

**Z-score is a measure of the overall model quality, and it was calculated by the Prosa-webserver (Wiederstein and Sippl, 2007). All the other parameters were calculated by Protein Structure Quality viewer implemented in the Schrodinger suite for molecular modeling.**

### Molecular Dynamics Simulations of the TMPRSS2 Models

Aimed to (1) understand the overall stability of the generated models, (2) to detect problematic or poorly modeled regions, and (3) to generate an ensemble of protein conformation for docking (Amaro et al., 2008; Sgrignani et al., 2009), we performed three 250 ns long MD simulations for each of the selected models. PROPKA calculations with the model from 5F8T indicated a positively charged catalytic histidine (His296) as the most probable state. However, considering that the same residue was predicted as His-εin the other two models and that this specific protonation state would be required to start the enzymatic reaction (Ishida and Kato, 2003), we simulated this specific residue in both protonation states.

**FIGURE 4:**
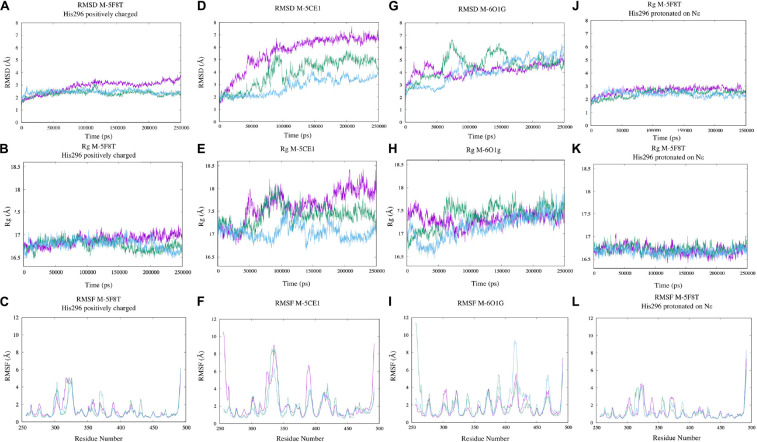
Analysis of the MD simulations of M-5F8T **(A–C)**, M-5F8T-His296 neutral **(D–F)**, M-5CE1 **(G–I)**, and M-6O1G **(J–L)**. The data from the three distinct simulations are depicted in different colors. RMSD, radius of gyration (Rg), and RMSF were calculated considering backbone atoms. RMSD, root mean square deviation; RMSF, root mean square fluctuation.

The simulation outputs were analyzed using consolidated observables such as RMSD, Rg, and the per-residue RMSF (Figure 4). This analysis highlighted a higher stability of M-5F8T with respect to M-5CE1 and M-6O1G. In particular, the simulations of M-5F8T always converged to a maximum RMSD of <3 Å from the starting model and Rg values similar to the starting one. Contrarily, M-5CE1 and M-6O1G showed continuously increasing RMSD and Rg profiles, suggesting that these models are less stable.

The RMSF profiles of M-5F8T and M-6O1G did not show anything relevant, substantially confirming the stability of M-5F8T. Contrarily, in M-5CE1, the protein region between positions 320 and 350, which contains the Tyr322 and Ser333 loop discussed before, was characterized by high RMSF values.

**FIGURE 5:**
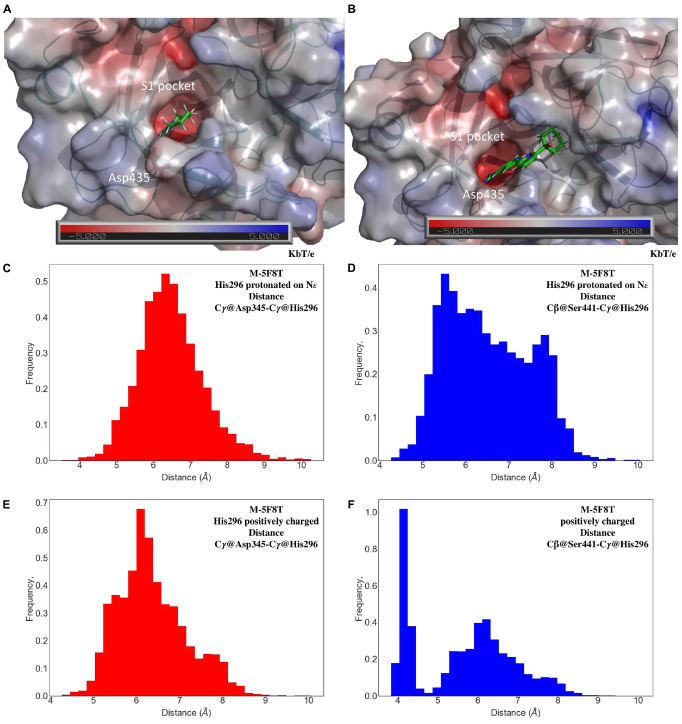
Representative conformations of the MD of M-5F8T **(A)** and M-5CE1 **(B)** with a focus on the interactions of PC1 and HPC with the S1 specificity pocket. The unit of electrostatic potential is kbT/*e* where kb, T, and *e* are the Boltzmann’s constant, absolute temperature, and the charge of an electron, respectively. Histogram analysis of the Cγ@Asp345-Cγ@His296 and Cβ@Ser441-Cγ@His296 distances in the MD simulations of M-5F8T with His296-ε **(C,D)** and His296 positively charged **(E,F)**. MD, molecular dynamics.

Small molecules in the catalytic site (PC1, HPC, 7SD, Figure 1) of the templates were preserved in the TMPRSS2 models, as the behavior of these molecules during MD simulations provides important hints about their quality and suitability to bind drugs. In the case of M-5F8T, the PC1 molecule remained in the S1 specificity pocket through a salt-bridge with Asp435 (Figure 5A). A similar behavior was also observed in the MD simulations of M-5CE1 (Figure 5B) for HCP. Contrarily, P4C rapidly dissociated from M-6O1G in all the simulations, probably because of the lack of a positively charged group docking the molecule to the S1 specificity pocket.

The good structural parameters (Table 3), the higher stability with respect to the M-5CE1 and M-6O1G (Figure 4), and the stable binding observed for PC1 in all the performed simulations suggested M-5F8T as the most reliable TMPRSS2 model. We, therefore, analyzed this model more deeply, focusing on the geometry of the catalytic triad (Asp345, His296, and Ser441). The analysis of distances between the three residues (Figures 5C–F) showed that this region of the protein remained stable during all the performed simulations. However, the system with a charged His296 adopted a conformation more similar to the starting model in which the Cγ@Asp345-Cγ@His296 and Cβ@Ser441-Cγ@His296 distances are 5.1 and 4.4 Å, respectively.

### Docking of Camostat and Nafamostat to TMPRSS2

As in the MD simulations, docking of camostat and nafamostat in M-5F8T was performed with His296 in two protonation states, that is, positively charged and protonated on *ε*.

**FIGURE 6:**
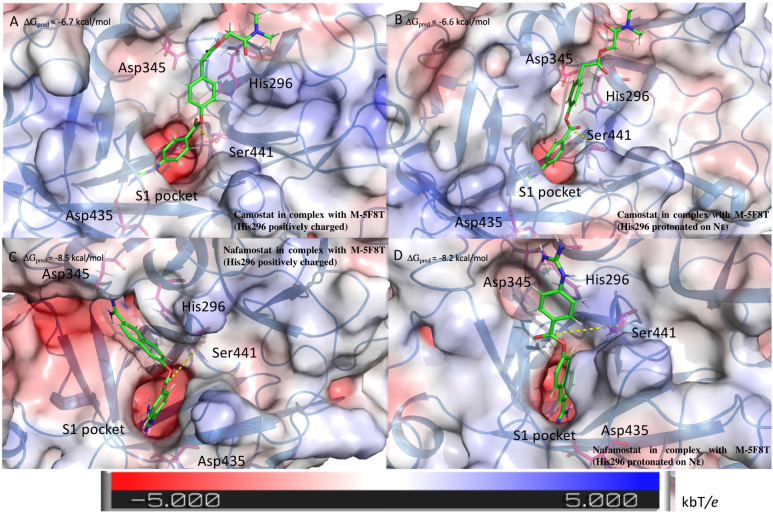
Structure of the complexes between TMPRSS2 and camostat **(A,B)** or nafamostat **(C,D)**. The pictures in the right and left columns refer to the docking calculation ran on M-5F8T considering His296 in its positively charged state or protonated on the ε nitrogen, respectively. The protein surface is colored in the function of the electrostatic potential according to the shown bar. The unit of electrostatic potential is kbT/*e* where kb, T, and *e* are the Boltzmann’s constant, absolute temperature, and the charge of an electron, respectively. The ΔG_pred_ values reported in the picture are the GlideScore values obtained from docking calculations.

The outcomes of these calculations (Figure 6) indicated that camostat adopts a similar binding mode irrespectively to the His296 protonation state. In particular, camostat places its guanidine group in the S1 specificity pocket where it forms a salt bridge with Asp435 orienting the other part of the molecule in the same direction.

Nafamostat is characterized by the presence of a guanidine group and one its isoster. Therefore, while one of these is placed in the S1 specificity pocket, the other forms different interactions depending on the His296 protonation state. In fact, in the model with ε protonated His296, the guanidine moiety forms a salt bridge with Glu299. On the contrary, in the model with the positively charged His296, the isosteric group directly binds Asp345, that is one of the members of the catalytic triad (Figure 6).

Interestingly, the binding score is not affected by the His296 protonation state. However, the predicted score is lower for nafamostat than camostat, which is in a qualitative agreement with literature that reports an IC50 value for nafamostat 10 times lower than for camostat (Yamamoto et al., 2016).

### Bromhexine Binding to TMPRSS2 Investigated by Microscale Thermophoresis

There have been discordant reports on the ability of BH to inhibit TMPRSS2. In fact, while the results of Lucas et al. (2014) appeared robust and convincing, a recent investigation by Hall and coworkers (Shrimp et al., 2020) concluded that BH is completely inactive as a TMPRSS2 inhibitor.

It is, however, important to consider that TMPRSS2 is a membrane protein with a peculiar and poorly understood activation mechanism. The purification of the active form of the enzyme, necessary for the inhibition tests, is thus extremely difficult. Furthermore, we noted that the protein quantity used in TMPRSS2 enzymatic assay is rarely reported (Meyer et al., 2013; Lucas et al., 2014), and that when reported (Shrimp et al., 2020), it extremely high (1 μM) with respect to the 1–2 nM concentrations used for other similar proteases (Hammamy et al., 2013; Ivanova et al., 2017). This suggests that the active species could be only a small fraction of the total protein, making it more difficult to observe a non-covalent weaker inhibition as that of BH. Thus, to better understand the existence and the strength of a BH/TMPRSS2 complex, we performed MST experiments.

MST is a recently developed biophysical technique that enables the investigation of molecular complexes measuring changes, upon binding, of the migration of target proteins in a laser-induced thermal gradient (Jerabek-Willemsen et al., 2011, 2014; Fassi et al., 2019).

The results of the MST experiments confirmed the BH/TMPRSS2 interaction with a Kd of 24 ± 13 μM (Figure 2A).

### Modeling the Interaction Between BH and TMPRSS2

Motivated by literature data (Lucas et al., 2014) and by the results of our MST experiments, we used computational methods to investigate the BH/TMPRSS2 interaction at an atomistic level.

A closer look at the chemical structure of BH revealed that it cannot form a covalent bond with the protein and, therefore, should have a different inhibition mechanism compared to camostat and nafamostat. We, therefore, performed docking calculations considering the catalytic site as a putative BH binding site. However, when simulated by MD, the ligand–protein complexes dissociated after few nanoseconds suggesting a low reliability of the obtained structures. This observation was validated by performing several simulations starting from slightly different initial poses of BH in the catalytic site obtained using runs with different grids (data not shown): inevitably, the BH-TMPRSS2 complex dissociates in few nanoseconds.

In their 2007 review, Laporte and Naesens (Laporte and Naesens, 2017) suggested that, because of its selectivity for TMPRSS2 over matriptase, trypsin, or thrombin, BH could exert its inhibitory effect binding to an allosteric site.

To better explore this hypothesis, we analyzed our models with Sitemap (Halgren, 2007, 2009), a computational tool already applied to the identification of allosteric sites (Sgrignani et al., 2014; Kots et al., 2017; Sanchez-Martin et al., 2020).

This analysis highlighted the existence of two putative drug binding sites (M-5F8T_site_1 and M-5F8T_site_2), for which a SiteScore value of >0.8 was obtained (Table 4). To note, while Site_1 describes a zone quite far from the active site, Site_2 includes also a part of the active sites Ser441 and His296.

**TABLE 4:** Results of the Sitemap analysis carried out on M-5F8T.

**Title**	**SiteScore**	**Dscore**	**Volume (Å^3^)**	**Residues**
M-5F8T_site_3	0.924	0.656	68.9	262, 263, 264, 265, 266, 267, 268, 270, 271, 272, 311, 312, 313, 314, 315, 316, 360, 384, 397
M-5F8T_site_2	0.892	0.907	201.3	274, 275, 277, 278, 279, 280, 281, 296, 300, 301, 302, 307, 308, 317, 384, 385, 386, 390, 391, 392, 393, 438, 439, 441
M-5F8T_site_1	0.863	0.872	279.2	369, 370, 371, 372, 373, 374, 376, 377, 403, 404, 405, 406, 407, 409, 413, 421, 422, 424, 425, 428, 429, 430, 469, 471, 476, 478, 479
M-5F8T_site_4	0.738	0.395	50.7	367, 368, 371, 372, 373, 375, 376, 447, 449, 454. 456, 478
M-5F8T_site_5	0.655	0.608	96.7	271, 291, 310, 311, 312, 325, 326, 327, 351, 355

In recent years, several algorithms for the prediction of putative allosteric sites (see also section “Materials and Methods”) have been developed. Therefore, to obtain a more comprehensive analysis, we carried out the same calculation using three additional algorithms (PARS, Deepsite, and FTMap). All these calculations confirmed the existence of Site_1, while Site_2 was identified by PARS and FTMap but not Deepsite.

Next, we docked BH in M-5F8T_site_1 and M-5F8T_site_2 and performed MD simulations of the complexes. While the simulations with BH bound to M-5F8T_site_2 resulted in a complex dissociating in the first 100 ns, the complex between BH and TMPRSS2 bound to M-5F8T_site_1 and remained stable for ∼1 μs. In fact, the drug remained close to the protein although it did not find a stable binding mode. Consequently, we performed two additional MD simulations to clarify this point. In the first control simulation, the ligand dissociated in the first 500 ns, while the second control simulation BH remained close to the protein surface without finding a stable binding mode, as in the first run. It should be also noted that in these simulations, while close to the protein surface, BH has a distance of ∼30 Å from the catalytic triad, making it difficult to imagine a direct effect on the catalytic activity from that position.

Summarizing the results of our simulations indicated that M-5F8T_site_1 and M-5F8T_site_2 are unsuitable to bind BH, leaving unsolved the question about the position of the BH allosteric site.

We, therefore, explored the possibility of the existence of a hidden allosteric site.

### The Role in Protein Activity of Free Isoleucine at the N-Terminal Side

The essential role for the enzymatic activity of the free isoleucine at the N-terminal side of TLPs has been previously reported (Stubbs et al., 1998; Huber, 2013; Meyer et al., 2013).

**FIGURE 7:**
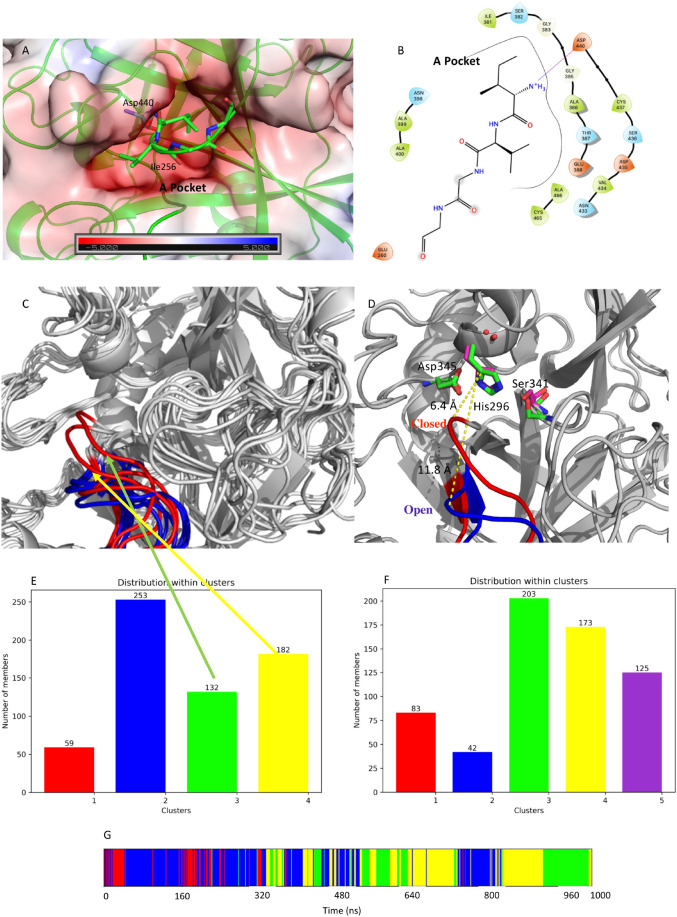
**(A)** Structure of the negatively charged cavity that hosts the N-terminal tail of the catalytically active TMPRSS2. The protein surface is colored by the electrostatic potential value calculated by the APBS plugin implemented in Pymol-2.3.4. The unit of electrostatic potential is kbT/*e* where kb, T, and *e* are the Boltzmann’s constant, absolute temperature, and the charge of an electron, respectively. **(B)** Scheme of the interactions between the N-terminal end and its binding site on the TMPRSS2 structure. **(C)** Conformations of the loop Gly462-Val473 in the representative structures from the identified clusters. The conformations from the simulation of apo-M-5F8T are shown in blue while those from the simulation of C-M-5F8T in red. **(D)** Comparison between the loop conformation assumed in the cluster3 of the C-M-5F8T MD (in red) and that of cluster3 of the M-5F8T MD (in blue). **(E)** Distribution of the C-M-5F8T conformations over the identified clusters. **(F)** Distribution of the M-5F8T conformations over the identified clusters. **(G)** Time evolution of the clusters obtained from the C-M-5F8T MD simulation. MD, molecular dynamics.

From visual inspection of M-5F8T, it can be seen that the N-terminal fragment of the protein occupies a negatively charged cavity (subsequently A-pocket, Figure 7) where the positively charged amino group of the N-terminal Ile256 forms a salt bridge with Asp440. Interestingly, Asp440 is contiguous to Ser441, one of the members of the catalytic triads, but oriented in a different direction.

To investigate the importance of this structural feature (i.e., the presence of free isoleucine at N-terminal site) for TMPRSS2, we generated a model of the enzyme deleting the first two residues at N-terminal (Ile256 and Val257) from M-5F8T (this model is subsequently indicated as C-M-5F8T) and performed an MD simulation for 1 μs.

Next, we compared the outputs of this simulation with an identical simulation of M-5F8T in its apo form.

Given its importance for the substrate recognition in TLPs and its structural proximity to the binding site, we first focused our analysis on the effect of the presence/absence of Ile256-Val257 on the structure of the S1 specificity pocket. Visual inspection of M-5F8T suggested that the Gly462-Val473 loop could regulate the access of the substrates to the S1 specificity pocket. We, therefore, analyzed the effects of the N-terminal truncation on the conformation of this protein region. Interestingly, we observed (Figure 7C) that, while in the simulation of the M-5F8T, the loop conserves a conformation similar to that adopted in the starting model; in the second part of the C-M-5F8T simulation, it moves closer to the catalytic triad (Figure 7D) occupying a position that should reduce the efficiency of both substrate recognition and catalysis.

**FIGURE 8:**
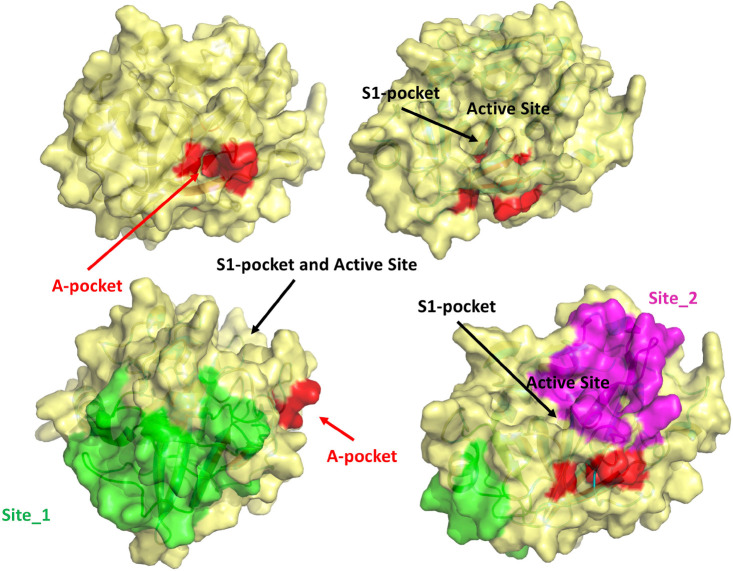
Visual summary of all the possible binding sites investigated in this study. The M-5F8T-C model is represented with different orientations to make clearer the reciprocal positions of the sites.

Taken together, these observations strongly suggest that the binding of the N-terminal tail into the A-pocket (Figures 7A,B, 8) is important to stabilize the structure of the TMPRSS2 active site and, in particular, of the S1 specificity pocket.

### Is the A-Pocket Relevant for Drug Design?

Considering the importance, highlighted by the previously discussed simulations, of the binding of the N-terminal tail into the A-pocket for the stability of the catalytic site, we performed some analysis to explore its druggability.

To this end, we first used Sitemap to analyze the C-M-5F8T model. This analysis showed that the cavity was made accessible by the deletion of the first two residues of M-5F8T and was highly suitable for drug binding, with a value of Sitescore of 0.93 over 1.00, where a druggable cavity should have SiteScore > 0.80. Next, we investigated if this pocket could be a suitable site for the BH binding.

Preliminary calculations (see section “Materials and Methods”) showed that the cavity was optimized for the binding of the N-terminal tail and not for the binding of a small molecule. We, therefore, first computed the optimal BH/TMPRSS2 binding pose using the IFD protocol implemented in Glide, followed by three MD independent simulations of 500 ns each.

The results of the docking calculations indicated the both (S)- and (R)-BH bind the A-pocket with a similar predicted affinity (−6.9 and −6.3 kcal/mol, respectively, Figures 2B–E).

From the structural point of view, both the molecules place the ring bearing the two bromine atoms in a cavity delimited by Gly363, Ile381, Ser382, Trp384, and Asp440. Moreover, the amino group in position 5 of the same ring establishes h-bond interactions with Asp440 and Gly383 in the case of (S)-BH and with Asn390 and Glu260 for (R)-BH. In both the structures, the positively charged amino group of BH electrostatically interacts with Glu260.

All MD simulations confirmed the stability of the complexes obtained from docking, with BH bound into the A-pocket for the entire simulation time.

Finally, we also performed a cluster analysis to verify the conformation of the Gly462-Val473 loop, which regulates the access to the S1 specificity pocket. This analysis (Supplementary Figure 1) clearly showed that the loop conserves a closed conformation in all the representative structures extracted from the simulations of (S)- and (R)-BH inside the A-pocket.

## Conclusion

TMPRSS2 is an exceptional and intriguing protein (Thunders and Delahunt, 2020), whose precise physiological function remains unknown. Despite this, it has been linked with several human diseases, such as prostate cancer, and has been shown to play a key role in viral infections.

In particular, the SPD of TMPRSS2 is critical for the priming of SARS-Cov-2 spike protein. This prompted us to investigate the interaction between TMPRSS2 various known drugs, using both computational and experimental methods.

While in the case of camostat and nafamostat, our computational studies confirmed that these two molecules bind to the active site of TMPRSS2 and form molecular adducts competent for the formation of covalent complexes; in the case of BH, our studies indicated that a competitive inhibition was unlikely.

On the other side, MST experiments confirmed a BH/TMPRSS2 interaction, leading us to ponder the hypothesis of an allosteric binding. We, therefore, used computer simulations to validate this hypothesis. The MD simulation confirmed that similar to other TLPs, the binding of a free isoleucine residue in the A-pocket is crucial to stabilize the catalytically competent active site conformation. Moreover, our calculations indicated that this cavity (Figure 8), fully accessible in the TMPRSS2 zymogen, is suitable to host BH or other more potent drugs that could be identified by virtual screening.

The study presented here provides further understanding of how the catalytic activity of TMPRSS2 can be modulated and new ways to develop more selective and potent antiviral treatments for current and future pandemics.

## Data Availability Statement

The raw data supporting the conclusions of this article will be made available by the authors, without undue reservation, to any qualified researcher.

## Author Contributions

JS designed the study, performed and analyzed simulations and experiments, and wrote and revised the manuscript. AC designed the study, analyzed the results of simulations and experiments, and wrote and revised the manuscript. Both authors contributed to the article and approved the submitted version.

## Conflict of Interest

The authors declare that the research was conducted in the absence of any commercial or financial relationships that could be construed as a potential conflict of interest.

## Footnotes

1 http://bioinf.uab.cat/cgi-bin/pars-cgi/pars.pl

2 https://playmolecule.org/deepsite

3 http://ftmap.bu.edu/login.php
