# Supplementary material for: Computational Identification of a Putative Allosteric Binding Pocket in TMPRSS2
Source: Front Mol Biosci. 2021 Apr 30;8:666626. doi: 10.3389/fmolb.2021.666626 (PMC8119889; doi:10.3389/fmolb.2021.666626)
Supplement: Supplementary file 1 [file Table_1.DOCX]

Supplementary Material

Analysis of the hydrogen bonds between the member of the catalytic triads

Asp345, His296 and Ser441

**M-5F8T His296 positively charged**

| **MD1** |  |  |  |  |  |
| --- | --- | --- | --- | --- | --- |
| **Acceptor** | **Donor-H** | **Donor** | **Frac** | **Average Distance** | **Average Angle** |
| ASP345@OD2 | HIP296@HD1 | HIP296@ND1 | 0.9513 | 2.6371 | 161.4847 |
| ASP345@OD1 | HIP296@H | HIP296@N | 0.8348 | 2.8176 | 166.7243 |
| ASP345@OD1 | HIP296@HD1 | HIP296@ND1 | 0.1030 | 2.8570 | 151.1883 |
| ASP345@OD2 | HIP296@H | HIP296@N | 0.0024 | 2.9081 | 166.1678 |
| SER441@O | SER441@HG | SER441@OG | 0.0024 | 2.9256 | 143.2661 |
| **MD2** |  |  |  |  |  |
| **Acceptor** | **Donor-H** | **Donor** | **Frac** | **Average Distance** | **Average Angle** |
| ASP345@OD1 | HIP296@HD1 | HIP296@ND1 | 0.5946 | 2.6600 | 160.4515 |
| ASP345@OD2 | HIP296@HD1 | HIP296@ND1 | 0.4876 | 2.6722 | 159.6078 |
| ASP345@OD2 | HIP296@H | HIP296@N | 0.2953 | 2.8398 | 163.0096 |
| ASP345@OD1 | HIP296@H | HIP296@N | 0.2809 | 2.8498 | 163.6817 |
| SER441@O | SER441@HG | SER441@OG | 0.0008 | 2.9284 | 144.3477 |
| **MD3** |  |  |  |  |  |
| **Acceptor** | **Donor-H** | **Donor** | **Frac** | **Average Distance** | **Average Angle** |
| ASP345@OD1 | HIP296@HD1 | HIP296@ND1 | 0.6536 | 2.6467 | 161.8587 |
| ASP345@OD2 | HIP296@H | HIP296@N | 0.5132 | 2.8222 | 165.3207 |
| ASP345@OD2 | HIP296@HD1 | HIP296@ND1 | 0.4054 | 2.6719 | 161.2997 |
| ASP345@OD1 | HIP296@H | HIP296@N | 0.2474 | 2.8306 | 165.9947 |
| SER441@O | SER441@HG | SER441@OG | 0.0040 | 2.9065 | 138.5462 |

**M-5F8T His296 protonated on Nε**

| **MD1** |  |  |  |  |  |
| --- | --- | --- | --- | --- | --- |
| **Acceptor** | **Donor-H** | **Donor** | **Frac** | **Average Distance** | **Average Angle** |
| ASP345@OD2 | ASP345@H | ASP345@N | 0.0439 | 2.7698 | 139.5089 |
| ASP345@OD1 | ASP345@H | ASP345@N | 0.0391 | 2.7742 | 139.6918 |
| ASP345@OD1 | HIS296@H | HIS296@N | 0.0128 | 2.8824 | 157.2922 |
| SER441@O | SER441@HG | SER441@OG | 0.0016 | 2.8962 | 139.7747 |
| ASP345@OD2 | HIS296@H | HIS296@N | 0.0008 | 2.9827 | 157.7611 |
| **MD2** |  |  |  |  |  |
| **Acceptor** | **Donor-H** | **Donor** | **Frac** | **Average Distance** | **Average Angle** |
| ASP345@OD2 | HIS296@H | HIS296@N | 0.0806 | 2.8753 | 160.5509 |
| ASP345@OD1 | HIS296@H | HIS441@N | 0.0567 | 2.8713 | 161.4785 |
| SER441@O | SER441@HG | SER441@OG | 0.0008 | 2.9111 | 137.957 |
| **MD3** |  |  |  |  |  |
| **Acceptor** | **Donor-H** | **Donor** | **Frac** | **Average Distance** | **Average Angle** |
| ASP345@OD1 | HIS296@H | HIS296@N | 0.0335 | 2.8745 | 160.1808 |
| ASP345@OD2 | HIS296@H | HIS296@N | 0.0231 | 2.8648 | 161.5487 |
| SER345@O | SER441@HG | SER441@OG | 0.0056 | 2.9444 | 145.3358 |

**M-5CE1**

| MD1 |  |  |  |  |  |
| --- | --- | --- | --- | --- | --- |
| Acceptor | Donor-H | Donor | Frac | Average Distance | Average Angle |
| ASP345@OD1 | HIS296@HD1 | HIS296@ND1 | 0.0846 | 2.7821 | 161.7914 |
| ASP345@OD2 | HIS296@HD1 | HIS296@ND1 | 0.0646 | 2.7551 | 155.5407 |
| ASP345@OD1 | HIS296@H | HIS296@N | 0.0263 | 2.8731 | 155.7519 |
| ASP345@OD2 | HIS296@H | HIS296@N | 0.0247 | 2.8357 | 158.8107 |
| SER441@O | SER441@HG | SER441@OG | 0.0072 | 2.9391 | 140.3635 |
| ASP345@OD2 | ASP345@H | ASP345@N | 0.0032 | 2.7294 | 140.9026 |
| ASP345@OD1 | ASP345@H | ASP345@N | 0.0032 | 2.7572 | 138.7182 |
| HIS296@O | HIS296@HD1 | HIS296@ND1 | 0.0016 | 2.7292 | 146.1677 |
| HIS296@NE2 | SER441@HG | SER441@OG | 0.0016 | 2.8163 | 150.2007 |
| HIS_41@ND1 | HIS_41@H | HIS_41@N | 0.0008 | 2.9222 | 137.4068 |
| **MD2** |  |  |  |  |  |
| **Acceptor** | **Donor-H** | **Donor** | **Frac** | **Average Distance** | **Average Angle** |
| ASP345@OD2 | HIS296@HD1 | HIS296@ND1 | 0.1453 | 2.7866 | 159.1709 |
| ASP345@OD1 | HIS296@HD1 | HIS296@ND1 | 0.1285 | 2.7560 | 153.5957 |
| ASP345@OD2 | HIS296@H | HIS296@N | 0.0798 | 2.8583 | 155.5383 |
| ASP345@OD1 | HIS296@H | HIS296@N | 0.0295 | 2.8577 | 155.1408 |
| HIS296@NE2 | SER441@HG | SER441@OG | 0.0223 | 2.8439 | 158.9044 |
| ASP345@OD1 | ASP345@H | ASP345@N | 0.0040 | 2.7227 | 137.2766 |
| ASP345@OD2 | ASP345@H | ASP345@N | 0.0032 | 2.8075 | 136.9327 |
| HIS296@ND1 | HIS296@H | HIS296@N | 0.0016 | 2.9774 | 141.9103 |
| SER441@O | SER441@HG | SER441@OG | 0.0008 | 2.9773 | 136.2651 |
| **MD3** |  |  |  |  |  |
| **Acceptor** | **Donor-H** | **Donor** | **Frac** | **Average Distance** | **Average Angle** |
| ASP345@OD1 | HIS296@HD1 | HIS296@ND1 | 0.3256 | 2.7842 | 163.0871 |
| ASP345@OD2 | HIS296@HD1 | HIS296@ND1 | 0.2107 | 2.7660 | 154.5454 |
| ASP345@OD1 | HIS296@H | HIS296@N | 0.0942 | 2.8849 | 159.5473 |
| ASP345@OD2 | HIS296@H | HIS296@N | 0.0271 | 2.8392 | 153.0133 |
| HIS296@NE2 | SER441@HG | SER441@OG | 0.0120 | 2.8639 | 160.8370 |
| HIS296@ND1 | HIS296@H | HIS296@N | 0.0048 | 2.9399 | 139.3701 |
| SER441@O | SER441@HG | SER441@OG | 0.0024 | 2.8792 | 149.1731 |
| ASP345@OD1 | ASP345@H | ASP345@N | 0.0008 | 2.8163 | 146.7376 |

**M-6O1G**

| **MD1** |  |  |  |  |  |
| --- | --- | --- | --- | --- | --- |
| **Acceptor** | **Donor-H** | **Donor** | **Frac** | **Average Distance** | **Average Angle** |
| ASP345@OD1 | HIS296@HD1 | HIS296@ND1 | 0.5044 | 2.8110 | 156.6555 |
| ASP345@OD2 | HIS296@HD1 | HIS296@ND1 | 0.4765 | 2.7966 | 153.8035 |
| ASP345@OD1 | HIS296@H | HIS296@N | 0.2482 | 2.8751 | 161.6896 |
| ASP345@OD2 | HIS345@H | HIS296@N | 0.1460 | 2.8649 | 160.1779 |
| HIS296@NE2 | SER441@HG | SER441@OG | 0.0088 | 2.8774 | 162.6080 |
| SER441@O | SER441@HG | SER441@OG | 0.0008 | 2.9276 | 141.5245 |
| **MD2** |  |  |  |  |  |
| **Acceptor** | **Donor-H** | **Donor** | **Frac** | **Average Distance** | **Average Angle** |
| ASP345@OD2 | HIS296@HD1 | HIS296@ND1 | 0.6890 | 2.7388 | 159.3167 |
| ASP345@OD1 | HIS296@H | HIS296@N | 0.3724 | 2.8541 | 155.0529 |
| ASP345@OD1 | HIS296@HD1 | HIS296@ND1 | 0.2967 | 2.7889 | 158.0965 |
| ASP345@OD2 | HIS296@H | HIS296@N | 0.0766 | 2.8499 | 156.2147 |
| SER441@O | SER441@HG | SER441@OG | 0.0622 | 2.8776 | 141.3811 |
| ASP345@OD1 | ASP345@H | ASP345@N | 0.0159 | 2.7728 | 140.5736 |
| HIS296@NE2 | SER441@HG | SER441@OG | 0.0152 | 2.8923 | 155.7669 |
| ASP345@OD2 | ASP345@H | ASP345@N | 0.0064 | 2.7734 | 137.9736 |
| **MD3** |  |  |  |  |  |
| **Acceptor** | **Donor-H** | **Donor** | **Frac** | **Average Distance** | **Average Angle** |
| ASP345@OD2 | HIS296@HD1 | HIS296@ND1 | 0.6102 | 2.7369 | 161.5812 |
| HIS296@NE2 | SER441@HG | SER441@OG | 0.2979 | 2.8419 | 160.3090 |
| ASP345@OD1 | HIS296@HD1 | HIS296@ND1 | 0.2476 | 2.7885 | 159.5559 |
| ASP345@OD1 | HIS296@H | HIS296@N | 0.1318 | 2.8587 | 159.5061 |
| ASP345@OD2 | HIS296@H | HIS296@N | 0.0511 | 2.8672 | 163.1887 |
| ASP345@OD2 | ASP345@H | ASP345@N | 0.0160 | 2.7788 | 138.4193 |
| ASP345@OD1 | ASP345@H | ASP345@N | 0.0112 | 2.7444 | 139.4750 |
| SER441@O | SER441@HG | SER441@OG | 0.0056 | 2.8710 | 142.0794 |

**
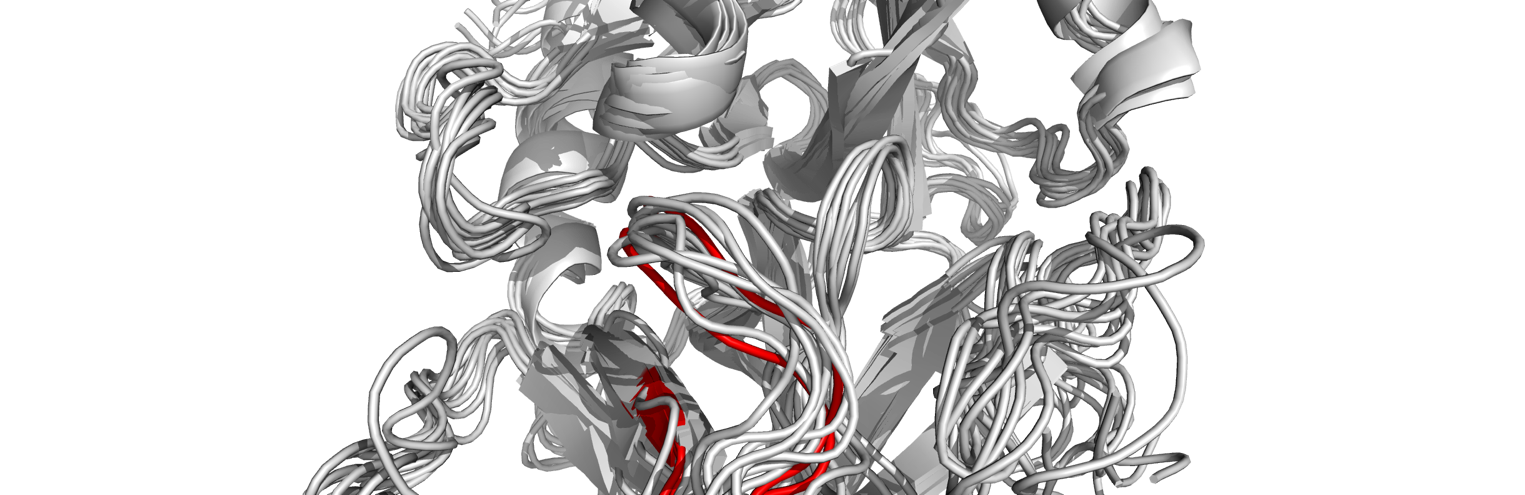
**

**Figure S1.** Conformation of the Gly462-Val473 loop in the representative clusters obtained from the analysis of the C-M-5F8T model in complex with BH in the A-pocket. The conformation of the loop we identified as closed in the simulations of the apo C-M-5F8T model is coloured in red.
